# Shared species of crocodilian trypanosomes carried by tabanid flies in Africa and South America, including the description of a new species from caimans, *Trypanosoma kaiowa* n. sp.

**DOI:** 10.1186/s13071-019-3463-2

**Published:** 2019-05-14

**Authors:** Bruno R. Fermino, Fernando Paiva, Laerte B. Viola, Carla M. F. Rodrigues, Herakles A. Garcia, Marta Campaner, Carmen S. A. Takata, Desie Sheferaw, John J. Kisakye, Agapitus Kato, Carlos A. G. S. Jared, Marta M. G. Teixeira, Erney P. Camargo

**Affiliations:** 1 Department of Parasitology, Institute of Biomedical Sciences, University of São Paulo, São Paulo, SP Brazil; 2 Biological Institute, Federal University of Mato Grosso do Sul, Campo Grande, Mato Grosso do Sul Brazil; 3 Department of Environment, Arcadis, São Paulo, Brazil; 4 Instituto Nacional de Ciência e Tecnologia, EpiAmo, Porto Velho, Rondônia Brazil; 5 Department of Veterinary Medicine, Hawassa University, Hawassa, Ethiopia; 6 Department of Zoology, Entomology and Fisheries Sciences, Makerere University, Kampala, Uganda; 7 Uganda Virus Research Institute, Entebbe, Uganda; 8 Instituto Butantan, Secretaria da Saúde, São Paulo, Brazil

**Keywords:** Tabanids, Tsetse flies, Crocodile, Caiman, Evolution, Taxonomy, Morphology, Transoceanic dispersal

## Abstract

**Background:**

The genus *Trypanosoma* Gruby, 1843 is constituted by terrestrial and aquatic phylogenetic lineages both harboring understudied trypanosomes from reptiles including an increasing diversity of crocodilian trypanosomes. *Trypanosoma clandestinus* Teixeira & Camargo, 2016 of the aquatic lineage is transmitted by leeches to caimans. *Trypanosoma grayi* Novy, 1906 of the terrestrial lineage is transmitted by tsetse flies to crocodiles in Africa, but the vectors of Neotropical caiman trypanosomes nested in this lineage remain unknown.

**Results:**

Our phylogenetic analyses uncovered crocodilian trypanosomes in tabanids from South America and Africa, and trypanosomes other than *T. grayi* in tsetse flies. All trypanosomes found in tabanids clustered in the crocodilian clade (terrestrial lineage) forming six clades: Grayi (African trypanosomes from crocodiles and tsetse flies); Ralphi (trypanosomes from caimans, African and Brazilian tabanids and tsetse flies); Terena (caimans); Cay03 (caimans and Brazilian tabanids); and two new clades, Tab01 (Brazilian tabanid and tsetse flies) and Kaiowa. The clade Kaiowa comprises *Trypanosoma kaiowa* n. sp. and trypanosomes from African and Brazilian tabanids, caimans, tsetse flies and the African dwarf crocodile. *Trypanosoma kaiowa* n. sp. heavily colonises tabanid guts and differs remarkably in morphology from other caiman trypanosomes. This species multiplied predominantly as promastigotes on log-phase cultures showing scarce epimastigotes and exhibited very long flagellates in old cultures. Analyses of growth behavior revealed that insect cells allow the intracellular development of *Trypanosoma kaiowa* n. sp.

**Conclusions:**

Prior to this description of *Trypanosoma kaiowa* n. sp., no crocodilian trypanosome parasitic in tabanid flies had been cultured, morphologically examined by light, scanning and transmission microscopy, and phylogenetically compared with other crocodilian trypanosomes. Additionally, trypanosomes thought to be restricted to caimans were identified in Brazilian and African tabanids, tsetse flies and the dwarf crocodile. Similar repertoires of trypanosomes found in South American caimans, African crocodiles and tabanids from both continents support the recent diversification of these transcontinental trypanosomes. Our findings are consistent with trypanosome host-switching likely mediated by tabanid flies between caimans and transoceanic migrant crocodiles co-inhabiting South American wetlands at the Miocene.

**Electronic supplementary material:**

The online version of this article (10.1186/s13071-019-3463-2) contains supplementary material, which is available to authorized users.

## Background

Trypanosomes are ubiquitous parasites of all classes of vertebrates, from fishes to mammals, including a few species parasitic in humans [1–4]. More than 70 trypanosome species have been described in reptiles (lizards, snakes, turtles and crocodilians) based on their morphology, and host of origin [2]. Until recently, *Trypanosoma grayi* Novy, 1906 [5, 6] and *Trypanosoma cecili* Lainson, 1977 [7] were the only trypanosomes described in African crocodiles and Brazilian caimans, respectively. Previous phylogenies, including *T. grayi* from tsetse flies and crocodiles [3, 8, 9] and trypanosomes from South American caimans [10–12] enabled the description of three additional species: *Trypanosoma terena* Teixeira & Camargo, 2013, *Trypanosoma ralphi* Teixeira & Camargo, 2013, and *Trypanosoma clandestinus* Teixeira & Camargo, 2016.

The genus *Trypanosoma* is composed of two major phylogenetic lineages, in line with the main habitats of their vertebrate hosts, the terrestrial and the aquatic lineage [3, 4]. Trypanosomes of fishes, anurans, chelonians, platypus and some lizards [3, 4, 10, 11, 13–19] plus *T. clandestinus* of caimans [12] cluster together in the aquatic lineage. Trypanosomes of land mammals, bats, birds, snakes and lizards, nest in the terrestrial lineage, together with the crocodilian trypanosomes *T. grayi*, *T. terena* and *T. ralphi* [4, 9–12, 20–23].

In Africa, trypanosomes of the terrestrial lineage occur in species of the family Crocodylidae: *Crocodylus niloticus* Laurenti, *Mecistops cataphractus* Cuvier, and *Osteolaemus tetraspis* Cope [5, 6, 12, 24]. In South America, trypanosomes are hosted by species of Alligatoridae (subfamily Caimaninae): *Caiman yacare* Daudin, *Caiman crocodilus* Linnaeus, *Melanosuchus niger* Spix, and *Paleosuchus trigonatus* Schneider [7, 10–12]. The extant species of the Crocodylinae and Caimaninae appeared on earth after an episode of mass extinction of crocodilians during the global cooling that extended from the mid-Miocene to the Pliocene [25–28]. The genus *Crocodylus* Laurenti originated in Australasia during the mid-Miocene (8–13 mya) and through transoceanic dispersal reached South America and Africa, although the exact dispersion route remains uncertain [26]. Fossil records from the mid- to late Miocene [27, 28] indicate that ancestors of caimans and crocodiles lived in sympatry in northern South America, a food-rich waterlogged ensemble of lowlands that gave origin to the contemporary Amazonia hydrographic system [29–31]. We previously hypothesised that the encounter of crocodiles with caimans created the opportunity for host-switching of trypanosomes, likely mediated by hematophagous invertebrates [10, 11]. The vectors of the African *T. grayi* are tsetse flies [5, 6, 32]. Leeches, that are common ectoparasites of crocodilians, are known vectors of *T. clandestinus* of caimans [12]. However, these aquatic leeches have never been reported to carry trypanosomes of the crocodilian clade. Thus, unknown hematophagous invertebrates other than leeches should be vectors of caiman trypanosomes of the terrestrial lineage. There is a range of contemporary hematophagous invertebrates that thrived on earth during the Cretaceous sharing habitats with crocodilians: leeches, ticks, mosquitoes, and flies of many families such as the Glossinidae, Psychodidae, Ceratopogonidae, and Tabanidae [33–36].

Tabanids cyclically transmit *Trypanosoma theileri* Laveran, 1902 of domestic and wild ruminants, carry other trypanosomes of ungulates, and possibly transmit trypanosomes to marsupials in Australia [1, 22, 37–41]. Many species of tabanids are generalist bloodsuckers, and some species feed preferentially on crocodiles, monitor lizards, snakes, chelonians and birds [42–46]. Given that some tabanid species prefer caiman blood as a food source [44], we searched for trypanosomes in tabanid flies captured near rivers and lakes inhabited by caimans or crocodiles. In this study, we report results from field surveys, morphological and behavioral features, and phylogenetic analyses of trypanosomes from tabanids, tsetse flies, caimans and crocodiles. Data obtained enabled the description of a new trypanosome species parasitic in tabanid flies and *Ca. yacare*.

## Methods

### Collection of tabanids and tsetse flies

**Fig. 1:**
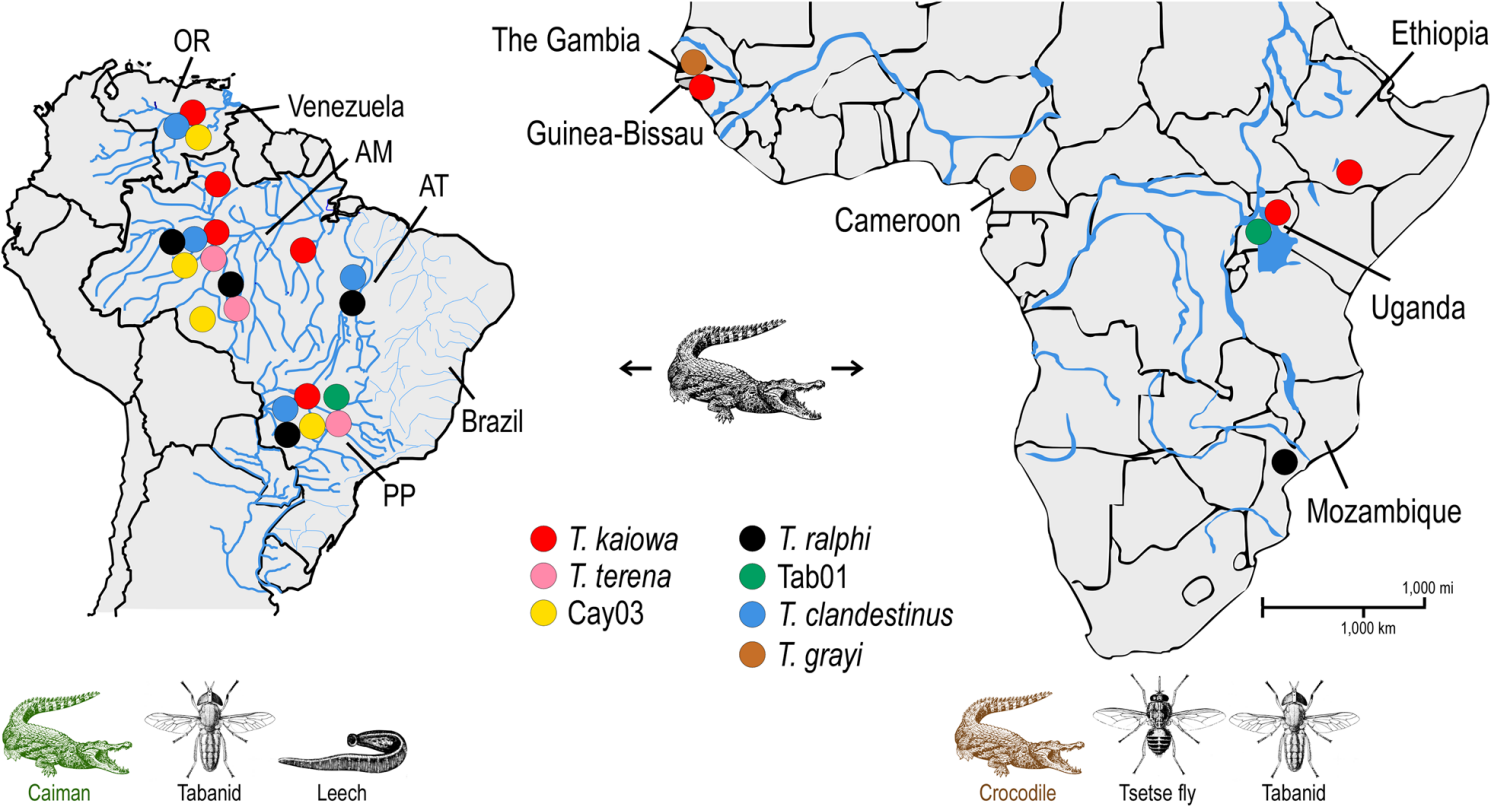
Geographical origin of crocodilian trypanosomes in South America and Africa. Trypanosomes were obtained from blood of caiman and crocodiles, and guts of tabanid and tsetse flies. Samples of *T. kaiowa* n. sp., *T. ralphi* and *Trypanosoma* sp. Tab01 were from Africa and South America; *T. terena, T. clandestinus* and Cay03 from South America; *T. grayi* from Africa. Hydrographic basins: OR, Orinoco; AM, Amazonas; AT, Araguaya-Tocantins; PP, Paraná-Paraguay

We collected tabanids while they were feeding on caimans (*Ca. yacare*) captured in the Miranda River, Mato Grosso do Sul state, in the Pantanal biome, which is a vast wetland in central Brazil and home to a considerable population of caimans (Fig. 1). Two adult caimans were captured [11, 12] and immobilized for about 3 h to serve as baits for tabanids. Tabanids were dissected and had their gut contents microscopically examined for trypanosomes according to procedures adopted for tsetse fly dissection detailed in a previous study [47]. Positive gut contents were inoculated into culture media and smeared on glass slides, which were fixed with methanol and stained with Giemsa.

We also collected tabanids near rivers and lakes inhabited by caimans or crocodiles. Tabanid flies were collected manually in the Amazonia biome, Tabajara Forest Reserve, Rondonia State, Brazil (8°56′S, 62°03′W), on the margins of the Ji-Paraná River (Fig. 1). In addition, tabanids and tsetse flies were caught on borders of the Zambezi River (Chupanga, district of Sofala) and Lake Jurema in the Gorongosa National Park in Mozambique, Lake Chamo in the Nech Sar National Park in Ethiopia, and Lake Edward in the Queen Elizabeth National Park in Uganda (Fig. 1). Flies from Amazonia and Africa could not be examined by microscopy and were preserved intact in ethanol. We confirmed the identification of selected specimens as representatives of the morphological diversity of tabanid, and tsetse flies by *cox*1 barcoding [48].

### Morphology and behavior of trypanosomes *in vitro*

Guts of tabanids caught in the Pantanal biome that were found to be microscopically positive for trypanosomes were seeded in cultures of Hi-5 insect feeder cells (*Trichoplusia* sp.) overlaid with TC100 (= Graceʼs) medium containing 10% FBS, and incubated at 25 °C [11, 12]. Flagellates were subsequently cultured in LIT (Liver Infusion Tryptose) medium supplemented by 10% FBS at 25 °C. The growth behavior of trypanosomes co-cultivated with Hi-5 cells was investigated in the supernatant and in cells attached to coverslips as we described previously [49]. Hi-5 cells in TC100 (~10^4^ cells/well) were seeded onto 24-well plates containing glass coverslips, incubated for 24–48 h, and then seeded with 10^5^ flagellates/well. After ~8 h, cultures were washed twice with PBS (phosphate buffered saline) and incubated with fresh TC100 medium. After 1, 3, 5, 7 and 10 days, cells were washed three times with PBS, and the cells fixed with methanol and stained with Giemsa.

Hi-5 cells seeded with trypanosomes were examined daily by inverted phase-microscopy. Examination of fixed flagellates stained with Giemsa in coverslips and mounted onto glass slides were performed by light microscopy. Both isolates TCC1611 and TCC2918 were evaluated regarding behavior in HI-5 cultures, and experiments with TCC2918 were repeated three times. Flagellates from the supernatant of Hi-5 cultures were fixed with methanol on glass slides and stained with Giemsa.

### Transmission (TEM) and scanning (SEM) electron microscopical analyses

For SEM, trypanosomes from log-phase Hi-5 cultures were washed three times with PBS, fixed in 2.5% glutaraldehyde plus 1% formaldehyde in 0.1 M cacodylate buffer (pH 7.2) at room temperature overnight, and processed using the protocols usually employed for trypanosomatids [11, 12, 17, 18, 20, 23, 49, 50]. Briefly, fixed flagellates were adhered to 0.1% poly-l-lysine-coated coverslips, post-fixed in the dark in 1% osmium tetroxide in 0.1 M cacodylate buffer, pH 7.2, for 30 min, dehydrated in an ascending ethanol series, dried in critical point drier equipment CPD 030 (Leica Microsystems, Wetzlar, Germany) and covered with gold in sputtering equipment SCD 050 (Leica Microsystems). A FEI Quanta 250 (FEI Company, Hillsboro, United States) was used for SEM. For TEM, trypanosomes were fixed and dehydrated as above for SEM, embedded overnight in epoxy resin, trimmed, and cut in thin sections (80–100 nm). Sections were stained with aqueous 2% uranyl acetate and lead citrate before being examined using a LEO 906E at 80 kV (Zeiss, Jena, Germany). Images were captured by a CCD camera MegaView III.

### PCR amplification, sequencing and phylogenetic analyses of *SSU* rRNA and *gGAPDH* sequences

DNA from caiman blood and gut contents of tabanid and tsetse flies preserved in ethanol were prepared as previously described [12, 47]. Briefly, blood and gut samples were centrifuged to remove ethanol, dried at 37 °C, resuspended in 200 µl of DIGSOL solution (containing proteinase K and RNAse), and incubated at 37 °C overnight. DNA was then precipitated with 400 μl of ammonium acetate (4 M), washed in ethanol, dried, and resuspended in 50–100 μl of TE.

DNA from caiman blood samples and the guts of tabanid and tsetse flies were submitted to nested-PCRs for amplification of V7V8 *SSU* rDNA (~900 bp) and *gGAPDH* (~680 bp) sequences, as previously described [12]. Sequences from 5–7 clones of each amplified sample were determined, screened for chimera by the RDP4 package, and those that were representatives of the whole genetic diversity were deposited in GenBank (Additional file 1: Table S1). For phylogenetic inferences, newly generated and published sequences were aligned with Clustal W and MUSCLE programs. We created three alignments: (i) A, consisting of V7V8 rDNA sequences of all trypanosomes found in crocodilians, including *T. clandestinus* and trypanosomes of the aquatic clade; (ii) B, including *gGAPDH* sequences, determined in the present and previous studies, restricted to trypanosomes of the clade crocodilian (several isolates from each clade), plus sequences of closely related trypanosomes from tapirs (Brazil) [21] and rhinoceros (Sumatra) available on GenBank; (iii) C, consisting of concatenated V7V8 *SSU* rDNA and gGAPDH sequences from isolates representative of each species/clade of the major crocodilian clade, and trypanosomes representative of other main clades within the terrestrial and aquatic lineages. Phylogenetic analyses were inferred by maximum likelihood (ML) and Bayesian inferences (BI) as described previously [4, 9, 11, 12, 18]. The ML analysis was performed using RAxML v8.2 [51]. Tree searches employed GTRGAMMA and proportion of invariable sites, model parameters were estimated in RAxML over the duration of tree search, and bootstrap supports estimated with 500 replicates. The BI analysis was conducted using MrBayes [52] with GTRGAMMAI; the first 25% of the trees was discarded as ‘burn in’.

## Results

### Tabanids from Brazil and Africa and tsetse flies harbour trypanosomes from the crocodilian clade

**Fig. 2:**
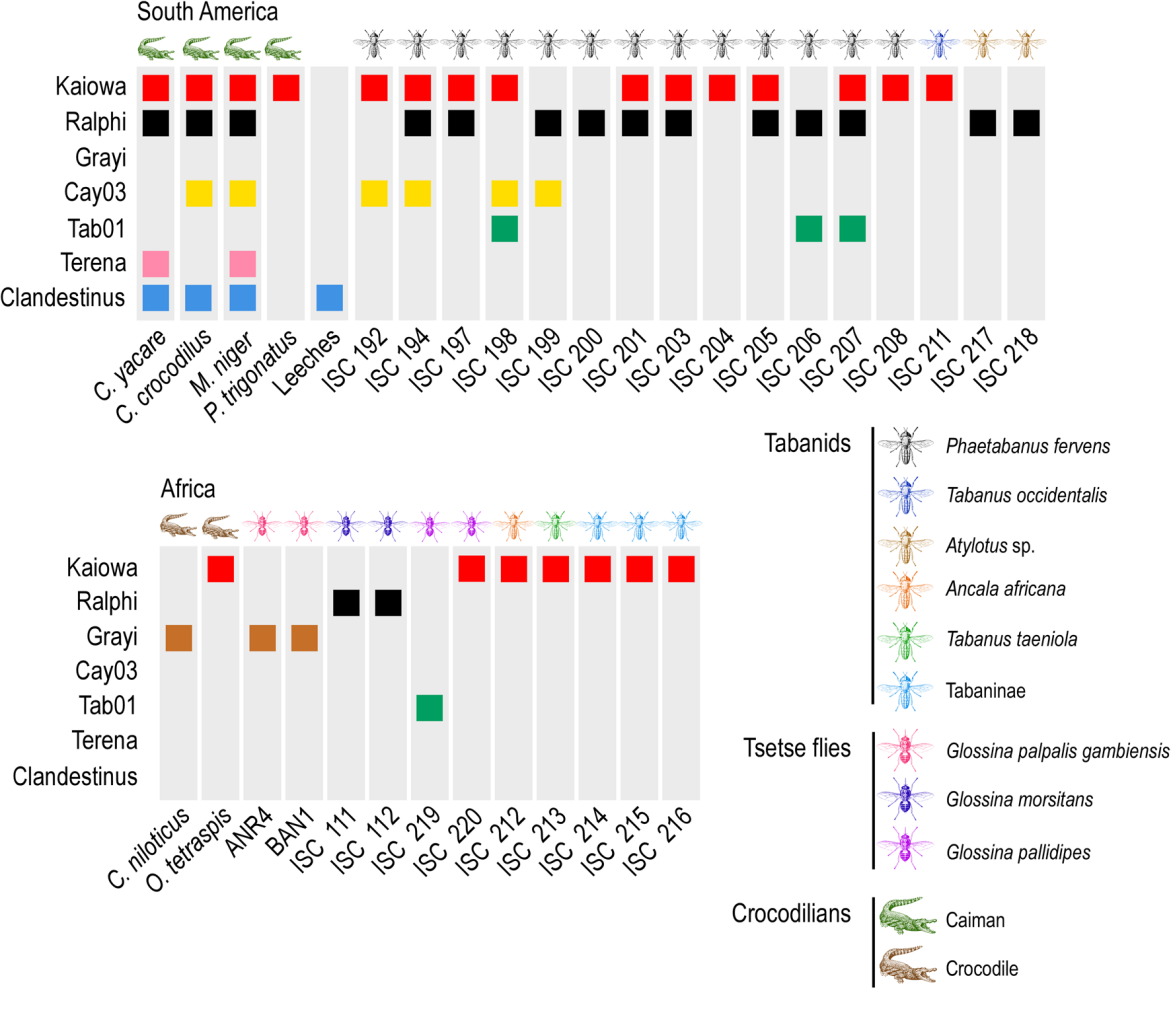
Trypanosomes in crocodilians, tabanids and tsetse flies in South America and Africa. Single and mixed infections identified by V7V8 *SSU* rRNA and/or *gGAPDH* barcodes of trypanosomes from the clades Kaiowa, Ralphi, Grayi, Cay03, Tab01, Terena and Clandestinus in individual blood samples from caimans (*Caiman yacare*, *Caiman crocodilus*, *Melanonosuchus niger* and *Paleosuchus trigonatus*), leeches (*Haementeria* sp.) and crocodiles (*Crocodylus niloticus* and *Osteolaemus tetrapsis*), and guts of tabanids (ISC 212-216) and tsetse flies (ANR4, BAN1, and ISC111, 112, 219, 220). In South America, *T. kaiowa*, *T. ralphi* and *Trypanosoma* sp. Cay03 were detected in tabanids and caimans, *T. terena* exclusively in caimans, *Trypanosoma* sp. Tab01 in tabanids, and *T. clandestinus* in caimans and leeches. In Africa, *T. kaiowa* n. sp. was identified in tabanids, tsetse flies and crocodiles, *T. ralphi* and *Trypanosoma* sp. Tab01 in tsetse flies, and *T. grayi* in tsetse flies and crocodiles

**Fig. 3:**
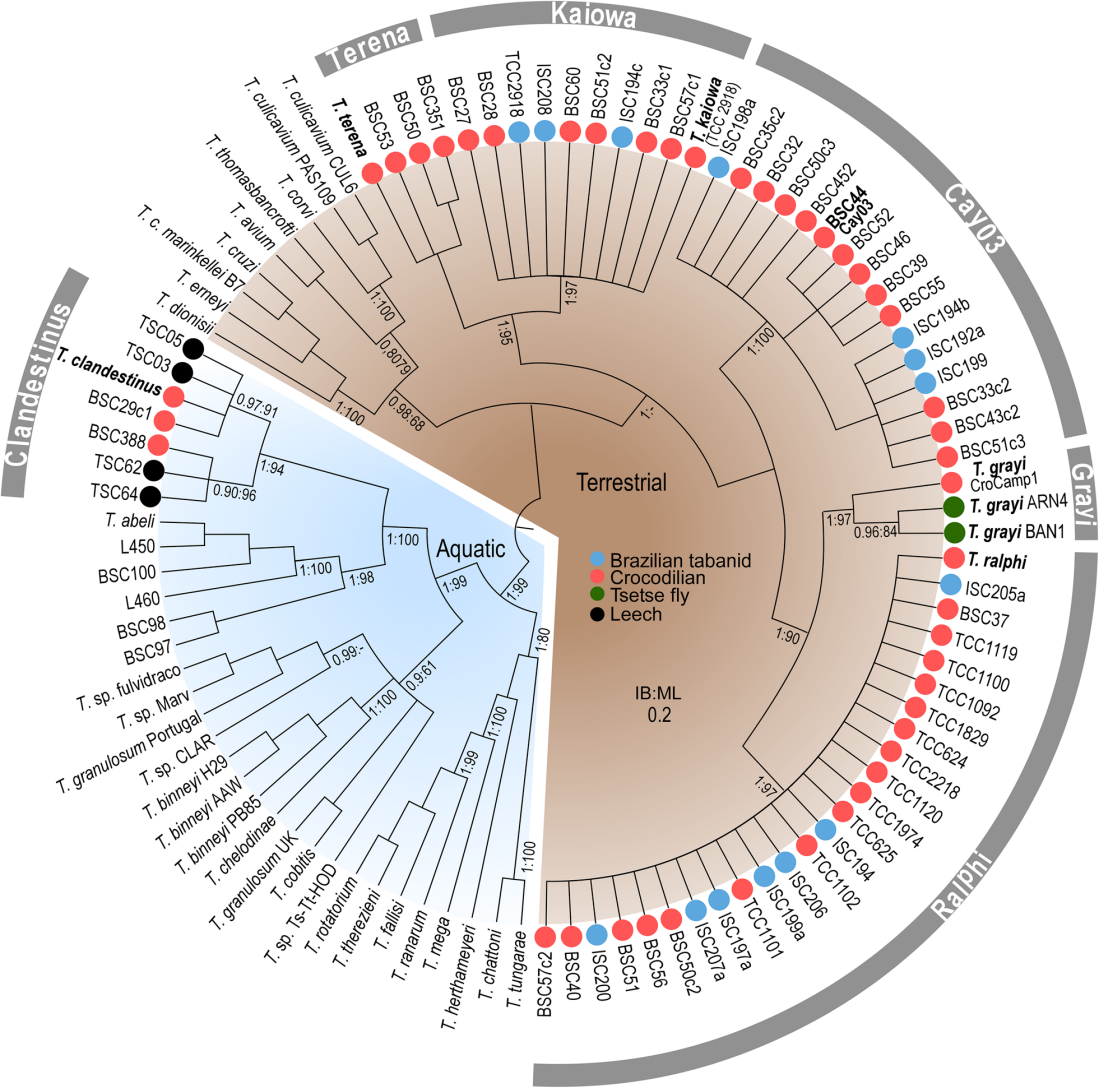
Phylogenetic analysis based on V7V8 *SSU* rRNA gene sequences of trypanosomes from caimans, crocodiles, tabanids and tsetse flies. Maximum Likelihood inference (903 characters, Ln = −4371.770725) supported the clades Ralphi, Kaiowa, Terena, Cay03 and Grayi in the terrestrial lineage. Trypanosomes of the aquatic lineages, including *T. clandestinus,* were used as outgroups. Numbers at the nodes (Bayesian Inference/Maximum Likelihood) represent the posterior probability > 0.8 and bootstrap support > 60%, respectively, derived from 500 replicates

**Fig. 4:**
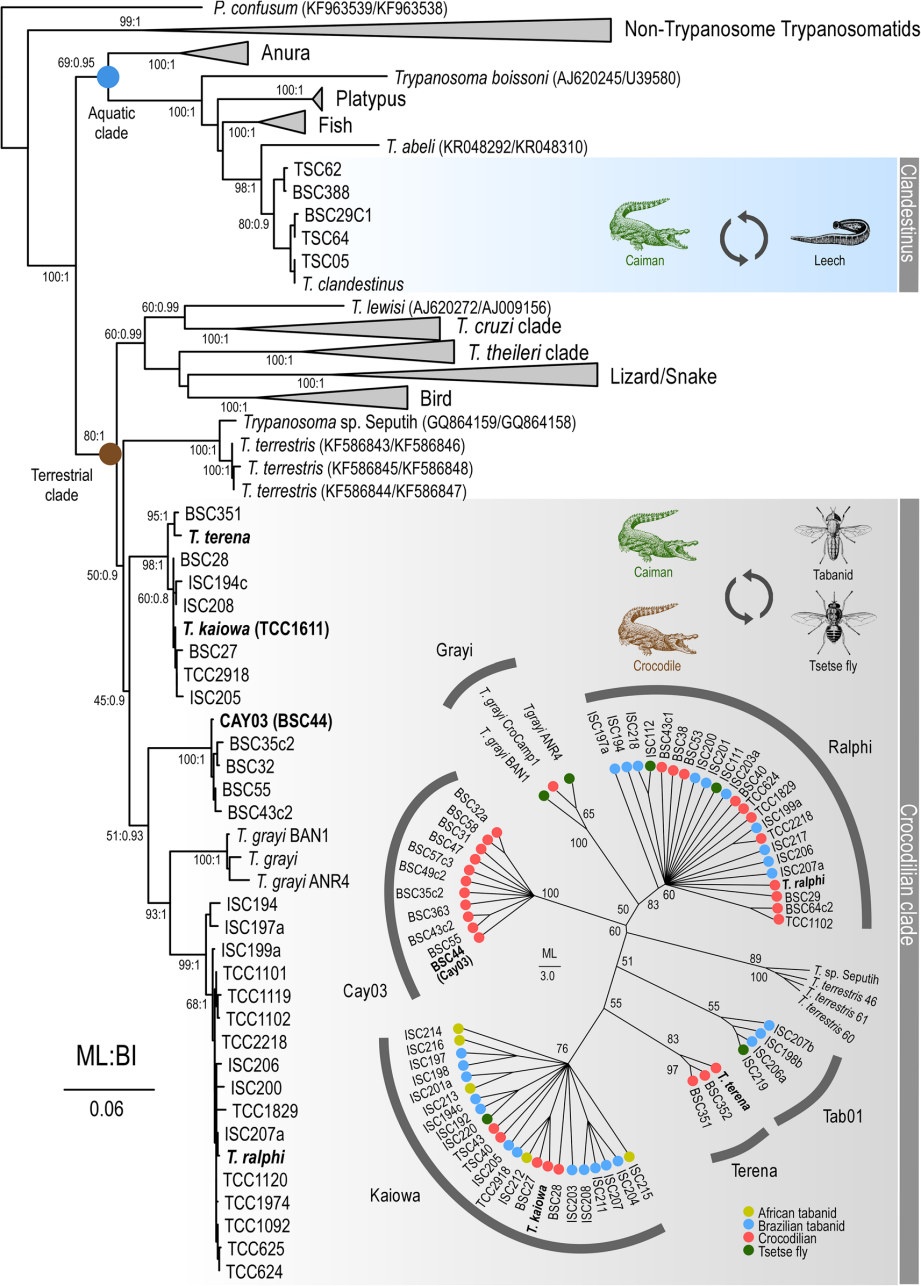
Phylogenetic tree (ML) inferred using concatenated *SSU* rRNA and gGAPDH gene sequences of trypanosomes from caimans, crocodiles, tabanids, and tsetse flies. The analyses were inferred by Maximum Likelihood (ML, 1720 characters, Ln = −17593.067085) and Bayesian Inference (BI). Trypanosomes of the crocodilian clade were distributed in the clades Ralphi, Terena, Cay03, Tab01, Kaiowa, and Grayi. Trypanosomes of both terrestrial and aquatic lineages were used as outgroups. Numbers at nodes (ML/BI) are bootstrap support > 50%, and Bayesian posterior probability > 0.8 derived from 500 replicates. Insert: phylogram (ML) using *gGAPDH* sequences. Numbers at nodes are bootstrap values derived from 500 replicates

Two species of tabanids, *Phaeotabanus fervens* Linnaeus (48 flies) and *Tabanus occidentalis* Linnaeus (3 flies), herein identified by *cox*1 barcoding, were caught while feeding on *Ca. yacare* captured in the Miranda River, in the Pantanal biome, Brazil (Fig. 1). Nineteen flies, all females, were positive for trypanosomes by microscopy of their gut contents, yielding an overall trypanosome prevalence of ~37%. We obtained *gGAPDH* and/or V7V8 *SSU* rDNA sequences of trypanosomes in 17 out of these 19 positive guts: 15 from *P. fervens* and two from *Ta. occidentalis* (Additional file 1: Table S1, Figs. 2, 3, 4). Most sequences were highly similar to those of known caiman trypanosomes, namely *T. terena*, *T. ralphi* and *Trypanosoma* sp. Cay03. However, sequences not identical to any previously known caiman trypanosomes were obtained from gut DNA samples from 10 *P. fervens* and one *Ta. occidentalis.* These sequences were distributed into two new clades: Kaiowa and Tab01 (Figs. 2, 3).

Barcode analysis revealed that mixed infections by more than one trypanosome were highly prevalent in the Brazilian tabanids (Fig. 2). In the Pantanal biome, 65% of tabanids held mixed infections. Single infections were detected in just six flies: four harbouring caiman trypanosomes, one harbouring a bird trypanosome, and another harbouring *T. theileri*. We never detected *T. clandestinus* in tabanid flies.

Tabanids (*Atylotus* spp.; 83 flies) from Amazonia showed a 40% infection rate, and *gGAPDH* sequences obtained from these flies revealed that they were infected with *T. ralphi* (Additional file 1: Table S1, Fig. 3). Sequences from *T. theileri* and bird trypanosomes (currently being characterized) were also obtained from these flies. Of the Ethiopian tabanids (*Tabanus taeniola* Palisot de Beauvois and *Ancala africana* Griffith & Pidgeon) 11 out of 33 flies yielded sequences of trypanosomes, five of which were highly similar to those of *T. kaiowa* n. sp. (Additional file 1: Table S1, Fig. 4). DNA from guts of 37 tabanids from Mozambique submitted to PCR-sequencing of *gGAPDH* yielded no crocodilian trypanosomes. Regarding tsetse flies, *gGAPDH* sequences herein analysed revealed the presence of *T. ralphi* in previously caught *Glossina morsitans morsitans* Wiedemann from Mozambique [47]. Also, in the present study, we detected *T. kaiowa* n. sp. and *Trypanosoma* sp. Tab01 in *Glossina pallidipes* Austen from Uganda (Additional file 1: Table S1, Figs. 3, 4).

From the gut contents of *P. fervens* (ISC203), we obtained one culture, which was cryopreserved as TCC2918. *SSU* rRNA and *gGAPDH* gene sequences from the isolate TCC2918 were identical to those of a previous isolate TCC1611 from *Ca. yacare* [11], which is being designed as the type-material of *Trypanosoma kaiowa* n. sp. (Figs. 3, 4).

### Phylogenetic inferences of trypanosomes from caimans, crocodiles, tabanid and tsetse flies

The comparison of V7V8 *SSU* rDNA barcode sequences (Fig. 3) of trypanosomes from caimans and crocodiles, and from tabanids and leeches collected over caimans, confirmed their separation in the major terrestrial and aquatic lineages [12]. *Trypanosoma* sp. Cay03, *Trypanosoma* sp. Tab01, *T. grayi*, *T. terena*, *T. ralphi* and *T. kaiowa* n. sp. clustered into a single and strongly supported phylogenetic assemblage: the crocodilian clade of the terrestrial lineage of trypanosomes. *Trypanosoma* sp. Seputih from Sumatran rhinoceros, inhabitants of marshy areas, and *Trypanosoma terrestris* Marcili, 2013 from semi-aquatic South American tapirs (Perissodactyla) [21] were found to be closely related to this clade (Figs. 3, 4). Interestingly, *Trypanosoma shawi* Nunes, 1987 [53] from capybaras, semi-aquatic Neotropical rodents that often share aquatic habitats with caimans and tapirs, was phylogenetically close to these trypanosomes (unpublished results). Nevertheless, corroborating the results of our previous study [12], *T. clandestinus* from caimans transmitted by aquatic leeches clustered with fish trypanosomes in the aquatic lineage.

Phylogenetic relationships inferred (ML and BI analyses) using either *gGAPDH* sequences or concatenated *SSU* rDNA and *gGAPDH* sequences strongly supported the partition of trypanosomes within the clade crocodilian into two main groups (Fig. 4). One phylogenetic group consists of the closely-related clades Kaiowa, Terena and Tab01 while the other consists of the clades Ralphi, Grayi (well-supported), and Cay03 (weakly supported) (Fig. 4). Each clade was led by one trypanosome species (separated by 3–6% gGAPDH sequence divergence), and each species showed small intraspecies divergence: 0.5% in *T. terena*; 0.6% in *T. ralphi*; 0.4% in *T. kaiowa*; and 2.2% in *T. grayi* from The Gambia and Cameroon. The relevant genetic divergence among the isolates from tsetse flies and *Crocodylus niloticus* forming the clade Grayi indicates the existence of different species/genotypes under the *T. grayi* designation. The relevant diversity within this clade may be related to the known genetic diversity of *C. niloticus* [54], and the large geographical distances between The Gambia and Cameroon (Fig. 1).

The separation of *T. kaiowa* n. sp. from the remaining species of crocodilian trypanosomes was confirmed by all analyses of the present study (Figs. 3, 4). It is worth noting that in our previous study [11], some isolates from caimans were included in the clade Terena despite their relevant divergences. The most divergent isolate included in this clade was TCC1611 from caiman, whose *gGAPDH* and *SSU* rDNA sequences showed to be identical to those of the isolate TCC2918 from a tabanid fly reported in the present study. These two isolates clustered with sequences obtained directly from guts of tabanid and tsetse flies, forming the just now well-resolved clade Kaiowa (Figs. 3, 4). The degrees of intra-clade sequence divergence and the phylogenetic positioning strongly support *T. kaiowa* n. sp. as the single species in the clade Kaiowa. The trypanosome from the African dwarf crocodile (BSC27) differs from *T. kaiowa* n. sp. by only 0.5% in gGAPDH sequences and, hence, should be regarded as a genotype of *T. kaiowa* n. sp. In contrast, large divergences (~8%) separated *T. kaiowa* n. sp. from *T. grayi*, so far restricted to African crocodiles.

### *Trypanosoma kaiowa* n. sp.: morphology and predicted life-cycle

Flagellates were not detected in blood smears of any caiman assuredly infected exclusively with *T. kaiowa* n. sp. Therefore, blood trypomastigotes of this species still required morphological characterization. However, *SSU* rDNA and *gGAPDH* sequences (15 cloned sequences from each isolate) from the isolates of caiman blood (TCC1611) and tabanid gut (TCC2918) were identical, indicating that we have obtained pure cultures of *T. kaiowa* n. sp. from both the vertebrate host, and its putative vector.

**Fig. 5:**
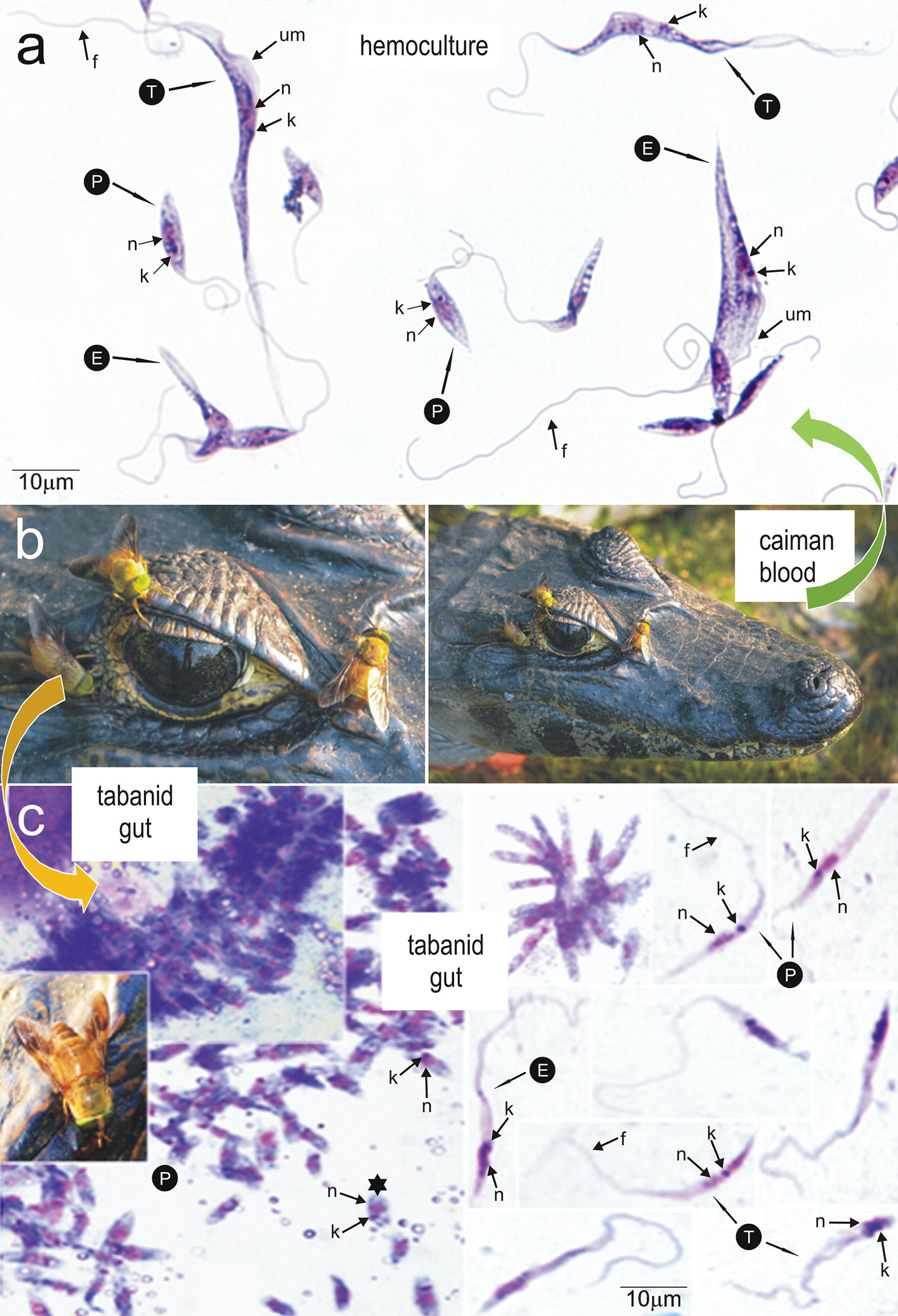
*Trypanosoma kaiowa* n. sp. predicted life-cycle. **a** Light microscopy of Giemsa-stained preparations of early hemocultures (BALB/LIT medium) from *Caiman yacare* naturally infected with *T. kaiowa* n. sp. showing large trypomastigote (T) and epimastigote (E) forms with noticeable undulating membrane, and small forms resembling promastigotes (P). **b** *Caiman yacare* and the tabanid *Phaeotabanus fervens* in the Pantanal, Brazil. **c** Clumps of flagellates adhered to a fragment of tabanid wall gut (yellow arrow), slim promastigotes (P), epimastigotes (E), and trypomastigotes (T) from gut contents of tabanids. *Abbreviations*: n, nucleus; k, kinetoplast; f, flagellum. *Scale-bars*: 10 µm

Early hemocultures of the isolate TCC1611 (Fig. 5a) from *Ca. yacare* (Fig. 5b) captured in the Pantanal, Brazil, exhibited long and pointed trypomastigotes and epimastigotes with perceptible undulating membranes. These morphological features differed from the large roll-shaped trypomastigotes with exuberant undulating membranes of *T. terena* and *T. ralphi* both in early hemocultures and in blood of caimans [10–12]. The small flagellates presented in TCC1611 hemocultures in general resembled promastigote forms (Fig. 5a).

We also analyzed the morphology of flagellates in gut content from the tabanid fly (*Phaeotabanus fervens)* (Fig. 5b, c) infected with the isolate TCC2918 of *T. kaiowa* n. sp. Microscopic examination of gut contents revealed large clumps of small dividing parasites attached to the gut walls (Fig. 5c). The masses of flagellates are sometimes so dense that it is impossible to distinguish separate individuals, except for small flagellates detached from the clumps of parasites (Fig. 5c). Flagellates found free in the lumen of tabanid guts were mostly slender promastigotes, but a few epimastigotes and forms with kinetoplast at the posterior end resembling trypomastigotes were also detected (Fig. 5c). Trypanosomes were restricted to the digestive tract of the examined tabanids. However, salivary gland infection in later stages of the tabanid infection still needs to be investigated.

### Development of *Trypanosoma kaiowa* n. sp. co-cultured with Hi-5 insect cells (25 °C)

**Fig. 6:**
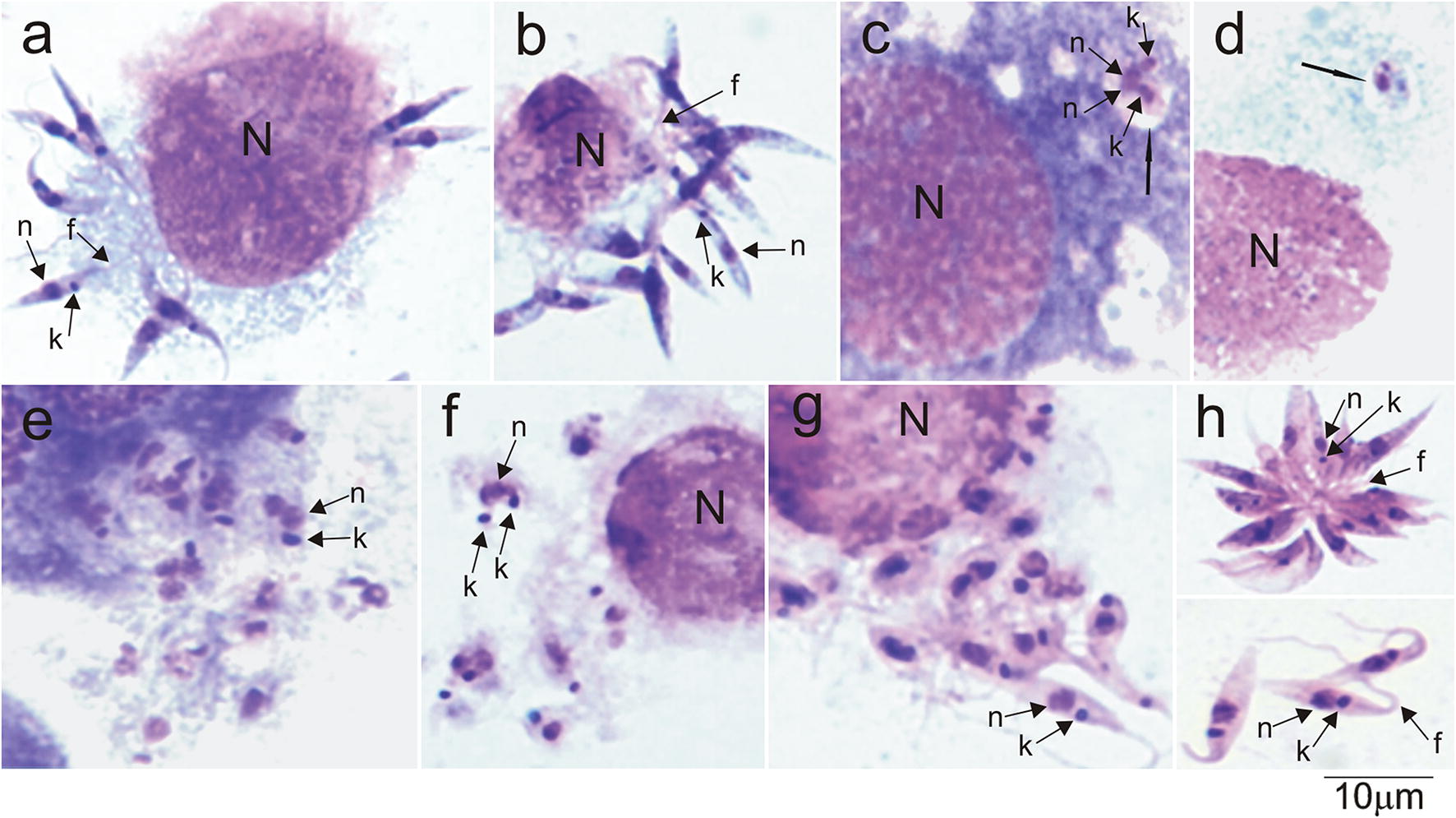
Growth behavioral of *Trypanosoma kaiowa* n. sp. co-cultivated with Hi-5 insect cells at 25 °C. Light microscopy of Giemsa-stained preparations showing: (**a**, **b**) flagellates adhered to Hi-5 cells by their flagella; (**c**, **d**) rounded flagellates within vacuoles (black arrows) in the cytoplasm of the insect cells; (**e**, **f**) agglomerates of dividing rounded forms; (**g**) rounded and promastigote forms, apparently released by host cells; (**h**) rosette and free short promastigotes in the supernatant of Hi-5 cells. *Abbreviations*: n, nucleus; k, kinetoplast; f, flagellum. *Scale-bar*: 10 µm

Light microscopy analyses of in Giemsa-stained preparations were conducted to evaluate the development of *T. kaiowa* n. sp. co-cultivated with a monolayer of Hi-5 insect cells in TC100 medium at 25 °C. In early cultures (24 h), inoculated promastigotes adhere to the surface of the insect cells by their flagella forming clusters of flagellates, and the flagellates likely invade the cells *via* the flagellum (Fig. 6a, b). After ~3 days, a few cells exhibited scarce rounded forms within vacuoles (Fig. 6c, d). However, 5–7 days cultures suggested that the parasites freely multiply in the cytoplasm of Hi-5 cells, and after ~7 days many cells show many rounded parasites (Fig. 6e, f). After 7–10 days, both rounded and short promastigote forms were, apparently, released by cells to the supernatant (Fig. 6g). The development of *T. kaiowa* n. sp. co-cultivated in Hi-5 cells was followed during ~20 days by observations of living (inverted phase microscopy), and fixed (Giemsa stained) cells. Growth behavior of *T. kaiowa* n. sp. in the supernatant of Hi-5 cultures was similar to cultures in LIT medium described below. Promastigotes multiply in the supernatant in general forming rosettes (Fig. 6h).

**Fig. 7:**
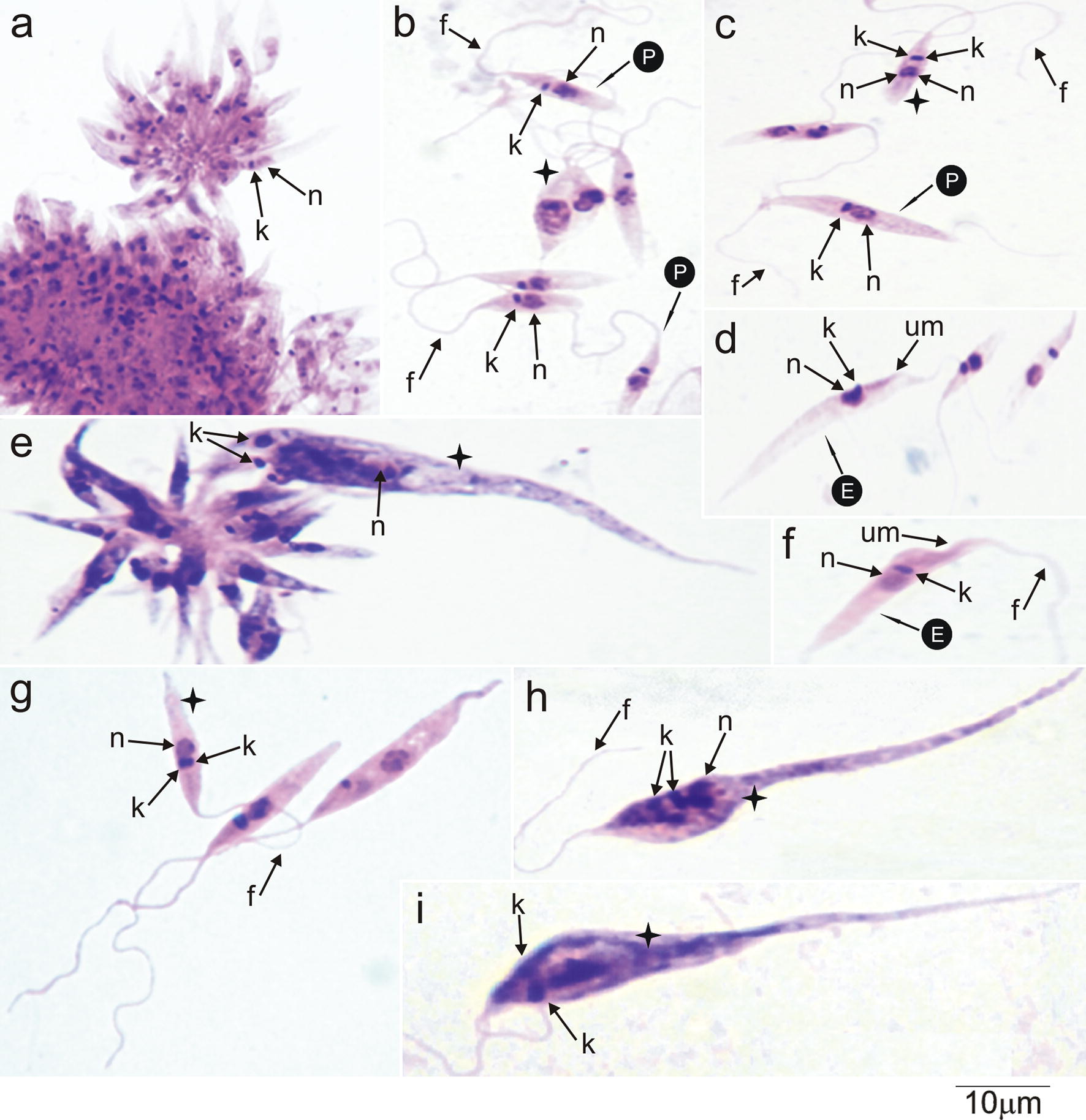
Development of *Trypanosoma kaiowa* n. sp. cultivated in LIT medium. **a** Rosettes of flagellates united by the flagella in log-phase cultures (3 days). **b**, **c** Detached free-swimming promastigotes (5–7 days). **d**–**f** Epimastigotes with undeveloped undulating membrane. **e** Rosette of large, irregular and pointed flagellates with a dividing flagellate still attached by the flagellum (10 days). **g** Long flagellates from stationary cultures varying in size and shape with long free flagellum. **h**, **i** Large flagellates with a long and pointed anterior extremity from old (15–20 days) cultures. *Abbreviations*: P, promastigote; E, epimastigote; n, nucleus; k, kinetoplast; f, flagellum; um, undulating membrane. Black crosses indicate dividing flagellates. *Scale-bar*: 10 µm

In early log-phase (2–3 days) of LIT cultures at 25 °C, *T. kaiowa* n. sp. forms small rosettes that quickly evolved to compact masses of numerous flagellates attached by their flagella (Fig. 7a). The flagellates gradually detach from the rosettes yielding abundant flagellates in the supernatant (~7 days). Log-phase trypanosomes lack perceptible undulating membranes, overall resembling small promastigotes (Fig. 7b, c). A few forms with lateral kinetoplast and an inconspicuous undulating membrane (characteristic of epimastigotes) were detected (Fig. 7d–f). Unexpectedly for stationary cultures, *T. kaiowa* n. sp. multiplied forming large rosettes of flagellates greatly varying in size (Fig. 7e) that gradually became very large flagellates with a pointed posterior extremity (Fig. 7h, i). A number of very long, often dividing flagellates, were observed in *T. kaiowa* n. sp. in old (> 15 days) cultures.

### Ultrastructural characterisation of *T. kaiowa* n. sp. by scanning and transmission electron microscopy

**Fig. 8:**
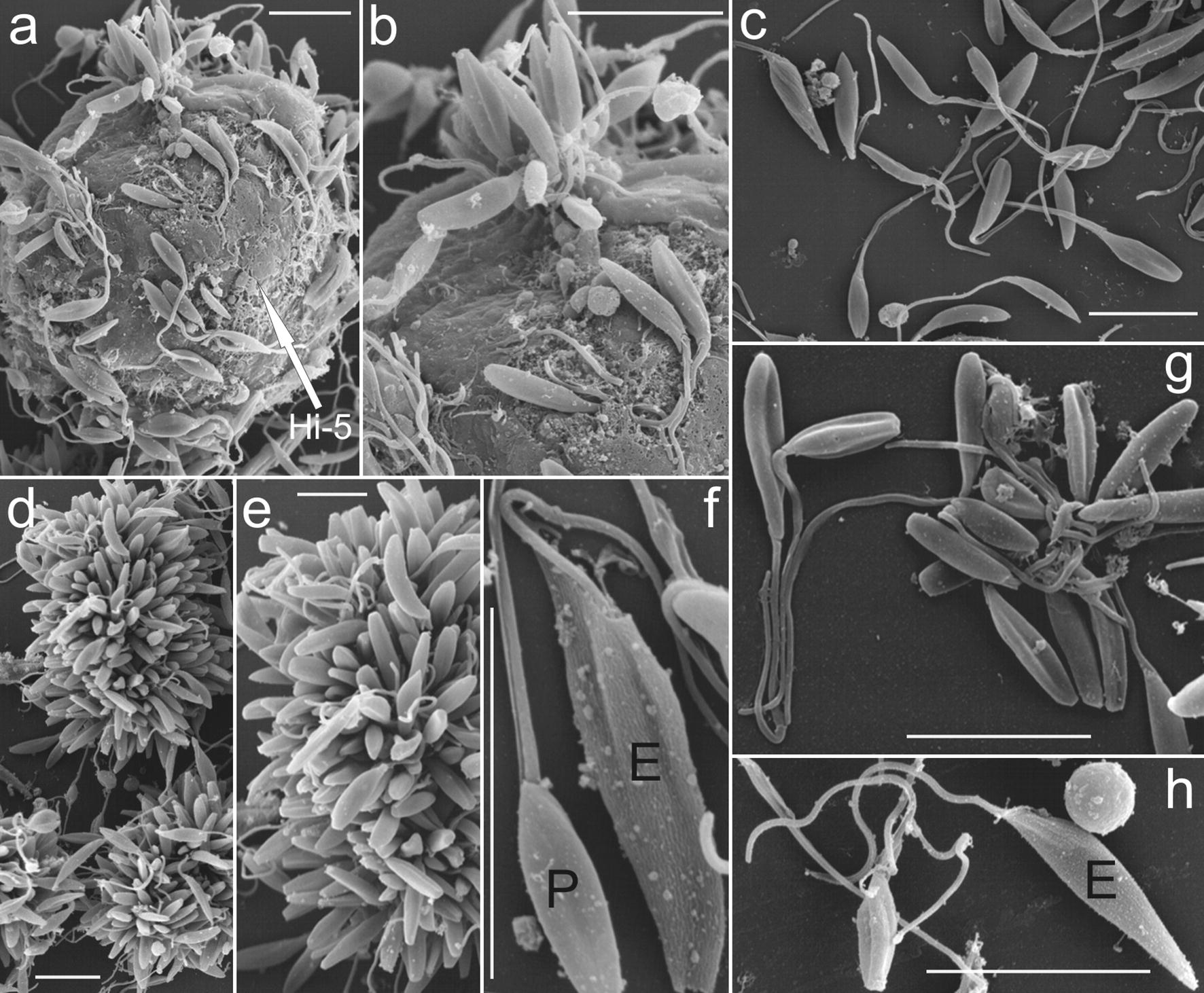
Scanning electron microscopy of *Trypanosoma kaiowa* n. sp. **a**, **b** Flagellates adhered to the membrane of Hi-5 insect cell (white arrow) cultivated in TC100 medium at 25 °C. **b** Flagellates apparently invading a Hi-5 cell *via* flagellum. **c P**romastigotes from culture supernatant. **d**, **e** Rosettes of promastigotes. **f** Promastigote (P) and epimastigote (E). **g** A clump of slender promastigotes exhibiting long flagella. **h** Epimastigote with an inconspicuous undulating membrane. *Scale-bars*: 10 µm

Scanning electron microscopy of *T. kaiowa* n. sp. flagellates co-cultivated with monolayers of Hi-5 cultures corroborated previous evidence from light microscopy of flagellates adhering to cell surface by their flagella, and apparently (Fig. 8a) penetrating HI-5 cells (Fig. 8b). Flagellates multiplied intensively (log-phase) either in supernatants (Fig. 7c) or adhered to coverslips forming large rosettes of flagellates attached by their flagella (Fig. 8d–e). Also confirming light microscopy, most flagellates resembled promastigote forms lacking a perceptible undulating membrane (Fig. 8c, f–h). A few pointed epimastigotes with an inconspicuous undulating membrane were detected in log-phase cultures of *T. kaiowa* n. sp. (Fig. 8f, h).

**Fig. 9:**
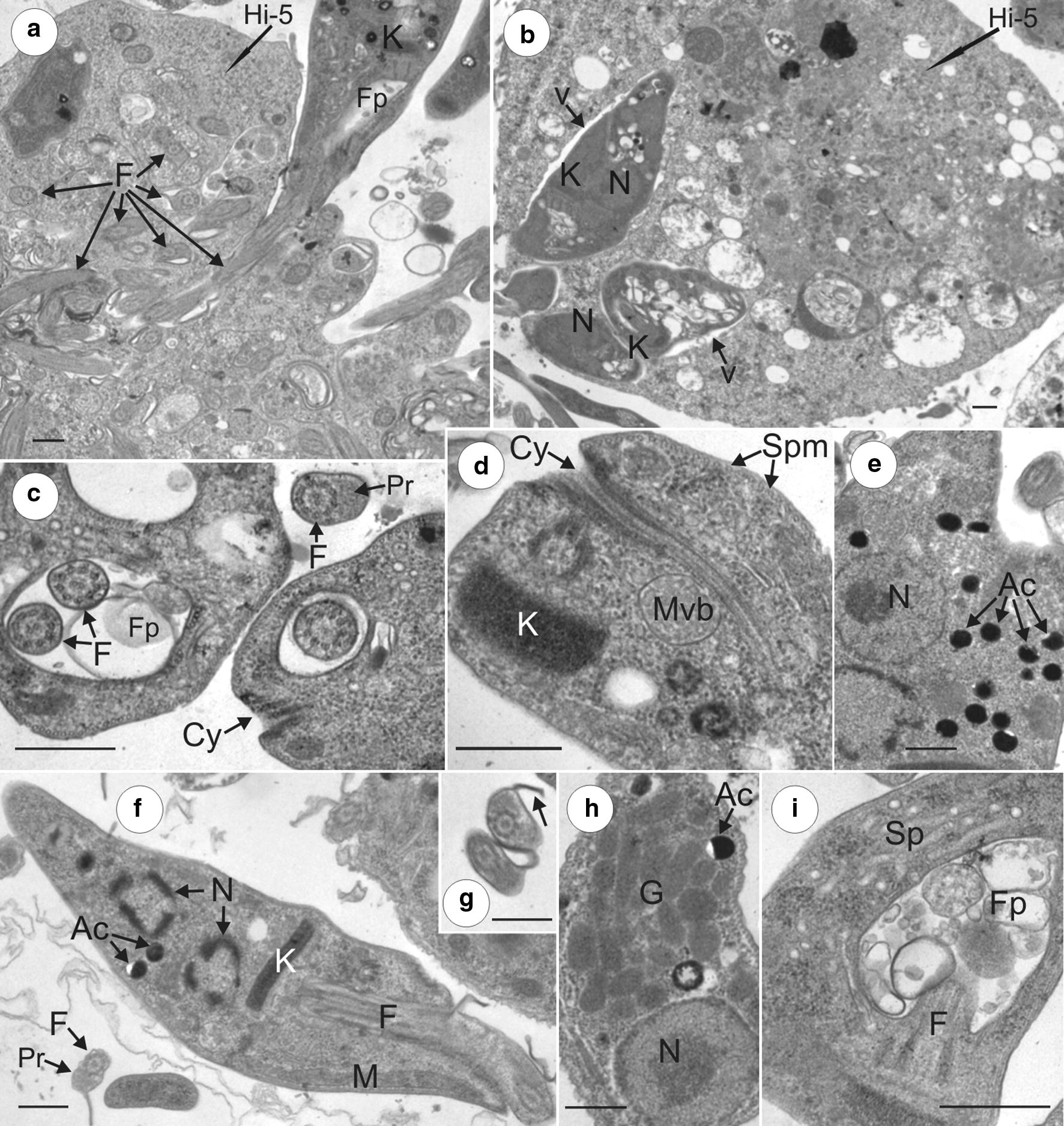
Ultrastructural features of *T. kaiowa* n. sp. revealed by TEM microscopy. **a** Flagellate invading Hi-5 cells *via* flagellum, and many transverse sections of the flagellum inside cell cytoplasm. **b** Small flagellates within tight vacuoles (indicated by black arrows) in the cytoplasm. **c** Transverse section of the flagellar pocket showing the typical structure of flagella, the absence of paraflagellar structure inside the flagellar pocket, prominent paraflagellar structure in free flagellum, and a small portion of the cytostome. **d** Longitudinal section of flagellate showing a deeply invaginated cytostome, compacted kinetoplast, and a multivesicular body probably containing viral particles. **e** Many acidocalcisomes in the cytoplasm. **f** Longitudinal sections of a dividing flagellate showing two nuclei. **g** Transverse section of flagella showing noticeable paraflagellar structure and lamella (black arrow). **h** Multiple glycosomes near the nucleus. **i** Network of tubules forming the spongiome adjacent to the flagellar pocket containing many vesicles. *Abbreviations*: N, nucleus; K, kinetoplast; Fp, flagellar pocket; F, flagellum; Cy, Cytostome; Pr, paraflagellar structure; Ac, acidocalcisomes; Spm, subpellicular microtubules; Mvb, multivesicular bodies; M, mitochondria; Sp, spongiome; v, vacuoles. *Scale-bars*: 0.5 µm

Transmission electron microscopy of log-phase *T. kaiowa* n. sp. co-cultivated with Hi-5 cultures revealed flagellates adhering to and, apparently, invading the insect cells (Fig. 9a, b). Transverse sections of a HI-5 cell showed multiple sections of one long internalized flagellum (Fig. 9a), and small flagellate within vacuoles in the cytoplasm of Hi-5 cells (Fig. 9b). The ultrastructural organization of *T. kaiowa* n. sp. flagellates from the supernatants of HI-5 cultures revealed all common organelles of trypanosomatids. However, a set of ultrastructural features of this new species merit to be highlighted: a cytostome (Fig. 9c, d) that deeply invaginates into the cytoplasm (Fig. 8d); large multivesicular bodies possibly containing virus particles (Fig. 9d); many acidocalcisomes (Fig. 9e); short kinetoplast (Fig. 9d, f); free flagella (portion outside the flagellar pocket) exhibiting well-developed paraflagellar structure (Fig. 9c, g), and noticeable lamella (Fig. 9g); multiple glycosomes near the nucleus (Fig. 9h), and well-developed tubules of the spongiome near the flagellar pocket that contains many vesicles (Fig. 9i).

### New species description

**Family Trypanosomatidae Doflein, 1951**

**Genus** ***Trypanosoma*** **Gruby, 1843**

***Trypanosoma kaiowa*** **Teixeira & Camargo n. sp.**

***Type-host***: *Caiman yacare* Daudin (Crocodylia: Alligatoridae).

***Other hosts***: *Caiman crocodilus* Linnaeus; *Paleosuchus trigonatus* Schneider; *Osteolaemus tetraspis* Cope.

***Type-locality***: Miranda River (19°57′S, 57°01′W) in the Pantanal, Mato Grosso do Sul, Brazil.

***Other localities***: Tabajara Reserve, Rondonia, Brazilian Amazonia; Lake Chamo, Ethiopia; Lake Edward, Uganda; Lagoas de Cufada Natural Reserve, Guinea Bissau.

***Type-material***: Hapantotype: the culture of the isolate TCC1611 from *Caiman yacare*. Paratypes: culture TCC2918 from the tabanid *Phaeotabanus fervens,* and caiman blood and tissue (liver) samples (BSC28; BSC60; TSC40, TSC43) and tabanid guts (ISC204, ISC208, ISC211) infected with *T. kaiowa* n. sp. The cultures of *T. kaiowa* n. sp are cryopreserved at the Trypanosomatid Culture Collection of the University of São Paulo, TCC-USP, registered in the World Culture Collection, Japan. Blood/liver samples of crocodilians were preserved in ethanol at BSC (blood sample collection) and TSC (tissue sample collection), and insect samples were preserved in ethanol at ISC (Insect Sample Collection). DNA from *T. kaiowa* n. sp. cultures, caiman blood samples and tabanid guts are all conserved at TCC-USP together with Giemsa-stained smears of cultures and tabanid guts infected with *T. kaiowa* n. sp.

***Invertebrate host (putative vector)***: *Phaeotabanus fervens* Linnaeus (Diptera, Tabanidae). Additional vectors: *Tabanus occidentalis* Linnaeus, *Ancala africana* Griffith & Pidgeon*, Tabanus taeniola* Palisot de Beauvois, and *Glossina pallidipes* Austen.

***Site in host***: Blood and liver of crocodilians; the digestive tract of tabanids and tsetse flies.

***Representative DNA sequences***: DNA sequences were deposited in the GenBank database as follows: KF546503 and MG680219 (V7V8 *SSU* rRNA gene); KF546517 and MG680244 (*gGAPDH* gene).

***ZooBank registration***: To comply with the regulations set out in article 8.5 of the amended 2012 version of the International Code of Zoological Nomenclature (ICZN) [55], details of the new species have been submitted to ZooBank. The Life Science Identifier (LSID) of the article is urn:lsid:zoobank.org:pub:5849C465-F9F8-4C4C-A331-95B745F4021C.

***Etymology***: “Kaiowá” refers to a formerly large Indian Nation occupying a vast area of Mato Grosso do Sul, Paraguay, and Argentina. The Kaiowá and Terena are closely related indigenous people, as closely related to each other are *T. terena* and *T. kaiowa* found in caimans.

### Description

Tabanid flies exhibited clumps of small oval-shaped flagellates attached to the gut walls, and free-swimming slender flagellates lacking perceptible undulating membranes resembling promastigotes, epimastigotes with inconspicuous undulating membranes, and trypomastigote forms. Early hemocultures exhibited long, wide and pointed trypomastigotes that evolved to rosettes of slender promastigotes. Measurements of log-phase promastigotes (LIT cultures) were 8.3–13 (10.3 ± 1.2) µm in length; 1.5–2.5 (2.0 ± 0.32) µm in width, and 12–25 (18.3 ± 3.6) µm of free flagellum. A few epimastigotes and very large and pointed flagellates were observed in old cultures. Light microscopy and ultrastructural features of all stages of *T. kaiowa* n. sp. are shown in Figs. 5, 6, 7 and 8

### Remarks

In our previous phylogenetic study, the isolate TCC1611 from *Ca. yacare* was placed in the clade Terena. However, this isolate was not considered a paratype of *T. terena* “because of relevant genetic divergences” (see [12]). In the present study, phylogenetic and morphological data confirmed that *T. terena* and the isolate TCC1611 (*T. kaiowa* n. sp.) are indeed distinct species. In addition, long and pointed trypomastigotes of *T. kaiowa* n. sp. differed from the roll-shaped trypomastigotes of *T. terena* and *T. ralphi* present in early hemocultures [10–12], and the predominance of promastigotes differentiates *T. kaiowa* n. sp. from these two species, both exhibiting epimastigotes with noticeable undulating membranes [11]. In addition, phylogenetic analyses also supported *Trypanosoma* sp. Cay03 and *Trypanosoma* sp. Tab01 as candidate new species. Unfortunately, they cannot yet be designated as new species because there are no valid holotypes for them: no cultures are available, and trypanosomes were not detected either in smears of caiman blood/tissue or tabanids single-infected with these trypanosomes.

## Discussion

The improvement of molecular approaches for the straightforward barcoding of Trypanosomatidae, including next-generation sequencing, has unveiling an increasing number of previously unidentified genera and species of trypanosomes in a wide range of vertebrate and invertebrate hosts [4, 9, 11–17, 19–23, 50, 56–62]. Recent descriptions of new species and genotypes has largely enriched the genetic diversity of the genus *Trypanosoma*, and drastically improved our understanding of their evolutionary histories, but are still far from counterbalancing the bias of studies towards pathogenic trypanosomatids in humans and livestock. In addition to many fieldwork difficulties and the permits required for capturing wild animals and collecting blood/tissue samples, additional challenges include the proper identification of vectors, the assessment of both host species and geographical ranges, investigation of life-cycles and developmental stages, and potential pathogenicity of any newly discovered species.

This study provides ecological, biological, behavioral and molecular evidence supporting tabanids as vectors of *T. kaiowa* n. sp. Our examination of tabanid guts revealed masses of small flagellates attached to the gut epithelium indicating that *T. kaiowa* n. sp. heavily colonizes tabanid guts. Similarly, during its development in naturally and experimentally infected tsetse flies, *T. grayi* also exhibited small flagellates attached by their flagella to the gut wall [6]. The development of *T. kaiowa* n. sp. in tabanids also appears to be like that reported for *Trypanosoma melophagium* Flu, 1908 and *Trypanosoma lewisi* Kent, 1880 in the gut epithelium of their vectors hippoboscid flies and fleas (exhibiting intracellular forms), respectively [1]. It is unknown whether, in natural infections, *T. kaiowa* n. sp. invades cells of tabanid guts similarly to what occurred herein within cultured Hi5 insect cells. All evidence indicates that tabanids are the vector of caiman trypanosomes, but confirmation still requires experimental infection of laboratory-raised caimans by tabanids, as has been done for *T. grayi* with tsetse flies and crocodiles [5, 6], and *T. clandestinus* with aquatic leeches and caimans [12].

A remarkable peculiarity of *T. kaiowa* n. sp. is the predominance of promastigote forms in tabanid guts and cultures, whereas typical epimastigotes are scarce in both cultures and tabanid flies. The predominance of promastigotes differentiates *T. kaiowa* n. sp. from *T. terena* and *T. ralphi* both exhibiting epimastigotes with noticeable undulating membranes [11]. In fact, predominance of promastigotes is rarely observed in cultures of mammalian trypanosomes [1], and whether this could be common in other trypanosomes remains to be investigated.

Our preliminary analyses demonstrated that *T. kaiowa* n. sp. can be co-cultivated at 34 °C with LLCMK_2_ mammalian cells, proliferates in the supernatants of cultures and, apparently, flagellates could enter the cells (data not shown). However, further investigations are necessary to verify a possible intracellular development of *T. kaiowa* n. sp. The ability of crocodilian trypanosomes to survive in mammals is an interesting question recently raised by the detection of *T. grayi* DNA in blood of cattle from Cameroon (Africa), where tsetse flies showed large prevalence rates of both *T. grayi* and trypanosomes of ungulates [60]. Moreover, trypanosomes sharing mammalian and reptilian hosts have been reported from naturally infected rodents, marsupials, snakes and lizards [61, 62].

Our molecular survey unveiled two species of crocodilian trypanosomes in tabanids, *T. kaiowa* n. sp. and *T. ralphi,* plus the still unnamed *Trypanosoma* sp. Cay03, and *Trypanosoma* sp. Tab01. All trypanosomes from caimans clustered with *T. grayi* forming the crocodilian clade of the terrestrial lineage. The only exception was *T. clandestinus* that remains the sole trypanosome species of caimans nested in the aquatic lineage of *Trypanosoma* [12]. So far, known vertebrate hosts of caiman trypanosomes are *Ca. yacare*, *Ca. crocodilus*, *M. niger* and *P. trigonatus*, which harbour single or mixed trypanosome infections throughout South American hydrographical basins [10–12]. Here, we demonstrated that different species of tabanids (*P. fervens*, *Ta. occidentalis* and *Atylotus* sp.) can harbour different trypanosomes of the crocodilian clade: *T. ralphi*, *T. kaiowa* n. sp., and the putative new species *Trypanosoma* sp. Cay03 and *Trypanosoma* sp. Tab01.

Findings of ‘South American’ trypanosomes in African tabanid and tsetse flies suggest an underestimated diversity of crocodilian trypanosomes on the African continent, before the present study thought to be restricted to *T. grayi*, and exclusively transmitted by tsetse flies [5, 6]. In a recent molecular epidemiological study of *Trypanosoma* spp. in the guts of tsetse flies from Cameroon, *T. grayi* was a highly prevalent species [60].

Here, tsetse flies were found infected with three trypanosomes: *T. ralphi* in Mozambique, and *T. kaiowa* n. sp. and *Trypanosoma* sp. Tab01 in Uganda. Heterogeneous sequences of *T. grayi* were obtained from tsetse flies from The Gambia, and crocodiles from Cameroon [63, 64]. The wide host diversity and broad geographical distribution of crocodilian trypanosomes are indicative of their lack of host restriction and spatial structure. Regarding the vectors of these trypanosomes, the scenario is different: leeches transmit trypanosomes of the aquatic lineage (*T. clandestinus*), while tabanid and tsetse flies transmit trypanosomes of the terrestrial lineage (crocodilian clade). The evolutionary history of trypanosomes relied on their phylogenetic relationships and eco-geographical and paleontological data of their vertebrate and invertebrate hosts. Although all data suggested an ancient and common origin for the trypanosomes of the crocodilian clade, the very close phylogenetic relationships among Neotropical and Afrotropical trypanosomes of crocodilians underline a recent diversification of all trypanosomes so far identified in this clade. There are no caimans in Africa and no African dwarf crocodiles in South America, but these animals share highly similar genotypes of the same species of trypanosomes. This current disjunct distribution of crocodilian trypanosome species is consistent with the transcontinental dispersal of crocodilians harboring trypanosomes [10–12, 26–28].

## Conclusions

To the best of our knowledge, data gathered in the present study provide the first evidence that tabanids can cyclically transmit trypanosomes to caimans and crocodiles in the Neotropic and Afrotropic. The description of *T. kaiowa* n. sp. in the present study relies on its phylogenetic positioning compared with other crocodilian trypanosomes. The description of *T. kaiowa* n. sp. is complemented by morphological and behavioural features of the flagellates in different growth conditions, and a predicted life-cycle in tabanid flies and caimans. The diversity and geographical dispersion of vertebrate and invertebrate hosts are also included in *T. kaiowa* n. sp. description. The close phylogenetic relationships of the crocodilian trypanosomes are in accordance with the transoceanic migration of crocodiles during the Miocene, and with the host-switching of trypanosomes between crocodiles and caimans. Our findings revealed that tabanid and tsetse flies are vectors of trypanosomes to crocodilians. Given that Glossinidae flies are absent from all continents other than Africa, adaptation to tsetse transmission would be secondary in the evolution of crocodilian trypanosomes, and tabanids, which are thought to have emerged during the Mesozoic (~140 mya), would be their ancestor vectors. However, this scenario relies on the contemporary geographical range of tsetse flies while records of *Glossina*-like fossils in the Nearctic and Palearctic at the Oligocene (30–40 mya) suggest that the ancestors of tsetse flies once flourished outside Africa, and possibly worldwide [36]. Data on crocodilian and tabanid trypanosomes from the Nearctic, Indomalayan and Australasian ecozones are still necessary for a better understanding of the evolutionary history and tracking the dispersion route of crocodilian trypanosomes.

## Additional file

**Additional file 1: Table S1.** Host species, geographical origin and *gGAPDH* and V7V8 *SSU* rRNA gene sequences of trypanosomes from crocodilians, tabanids and tsetse flies.

## Abbreviations

TCC: Trypanosomatid Culture Collection of the University of São Paulo, SP, Brazil
*SSU* rRNA: small subunit of ribosomal RNA
*gGAPDH*: glycosomal glyceraldehyde-3-phosphate dehydrogenase
PCR: polymerase chain reaction
Hi-5: insect (*Trichoplusia* sp.) cell
