# Supplementary material for: Shared species of crocodilian trypanosomes carried by tabanid flies in Africa and South America, including the description of a new species from caimans, Trypanosoma kaiowa n. sp
Source: Parasit Vectors. 2019 May 14;12:225. doi: 10.1186/s13071-019-3463-2 (PMC6515670; doi:10.1186/s13071-019-3463-2)
Supplement: Supplementary file 1 — Additional file 1: Table S1. Host species, geographical origin and gGAPDH and V7V8 SSU rRNA gene sequences of trypanosomes from crocodilians, tabanids and tsetse flies. [file 13071_2019_3463_MOESM1_ESM.docx]

**Additional file 1: Table S1**

**Host species, geographical origin and gGAPDH and V7V8 SSU rRNA gene sequences of trypanosomes from**

**crocodilians, tabanids and tsetse flies.**

| ***Trypanosoma***  **Sample^a^** | **Host** | **Country/**  **River Basin** | **GSP** | **GenBank accession number** | |
| --- | --- | --- | --- | --- | --- |
|  |  |  |  | **gGAPDH** | **V7V8 SSU rRNA** |
| **Clade Kaiowa** | | | | | |
| *T. kaiowa* n. sp. (TCC 1611) | *Caiman yacare* | BR/PP | 19°57’S 57°01’W | KF546517 | KF546503 |
| TCC 2918 | *Phaeotabanus fervens* * | BR/PP | 19°57’S 57°01’W | MG680244 | MG680219 |
| BSC 27 | *Osteolaemus. tetraspis* | GB/- | 11°60´N 15°04’W | KF546505 | MH718308 |
| BSC 28 | *Caiman crocodilus* | VE/OR | 6°83´N 67°69´W | KF546504 | KF546518 |
| BSC 33c1*, 51c2*, 57c1* | *Melanosuchus niger* | BR/AM | 2°19’S 65°70’W |  | KP768290-2 |
| BSC 60 | *Caiman crocodilus* | BR/AM | 7°27’S 64°80W |  | KP768293 |
| ISC 192 | *Phaetabanus fervens* * | BR/PP | 19°57’S 57°01’W | MG680233 |  |
| ISC 194c | *Phaetabanus fervens* * | BR/PP | 19°57’S 57°01’W | MG680234 | MG680220 |
| ISC 197,198, 201a, 203-205, 207 | *Phaetabanus fervens* * | BR/PP | 19°57’S 57°01’W | MG680235-41 |  |
| ISC 208 | *Phaetabanus fervens* * | BR/PP | 19°57’S 57°01’W | MG680242 | MG680221 |
| ISC 211 | *Tabanus occidentalis* * | BR/PP | 19°57’S 57°01’W | MG680243 |  |
| ISC 212 | *Ancala africana* ^#^ | ET/- | 5°48'N 37°32'E | MH716216 |  |
| ISC 213 | *Tabanus taeniola* ^#^ | ET/- | 5°48'N 37°32'E | MH716217 |  |
| ISC 214, 215, 216 | Tabaninae ^#^ | ET/- | 5°48'N 37°32'E | MH716218-20 |  |
| ISC 220 | *Glossina pallidipes* ^§^ | UG/- | 0°01'S 30°11'E | MH716224 |  |
| TSC 40 | *Paleosuchus trigonatus* | BR/AM | 3°80’N 61°73’W | KP768272 |  |
| TSC 43 | *Caiman crocodilus* | BR/AM | 3°20’S 51°86’W | KP768273 |  |
| **Clade Terena** | | | | | |
| *T. terena* (TC C610) | *Caiman yacare* | BR/PP | 19°57’S 57°01’W | EU596252 | EU596256 |
| BSC 50*, 53 | *Melanosuchus niger* | BR/AM | 2°19’S 65°70’W |  | KF546519-20 |
| BSC 351, 352 | *Melanosuchus niger* | BR/AM | 7°27’S 64°80W | MG680231-2 | MH718309 |
| **Clade Ralphi** | | | | | |
| BSC 29* | *Caiman yacare* | BR/PP | 19°57’S 57°01’W | KF546515 |  |
| BSC 37 | *Melanosuchus niger* | BR/AM | 2°19’S 65°70’W |  | KP768294 |
| BSC 38 | *Melanosuchus niger* | BR/AM | 2°19’S 65°70’W | KP768274 |  |
| BSC 40 | *Melanosuchus niger* | BR/AM | 2°19’S 65°70’W | KP768275 | KP768295 |
| BSC 42, 43c1* | *Melanosuchus niger* | BR/AM | 2°19’S 65°70’W | KP768276-7 |  |
| BSC 50c2* | *Melanosuchus niger* | BR/AM | 2°19’S 65°70’W |  | KP768296 |
| BSC 51* | *Melanosuchus niger* | BR/AM | 2°19’S 65°70’W |  | KF546524 |
| BSC 53 | *Melanosuchus niger* | BR/AM | 2°19’S 65°70’W | KP768278 |  |
| BSC 56 | *Melanosuchus niger* | BR/AM | 2°19’S 65°70’W |  | KF546525 |
| BSC 57c2* | *Melanosuchus niger* | BR/AM | 2°19’S 65°70’W |  | KP768297 |
| BSC 64c2* | *Caiman crocodilus* | BR/AT | 7°32’S 49°22’W | KF546516 |  |
| ISC 111, 112 | *Glossina morsitans* ^§^ | MZ/- | 18°51'S 34°29'E | MH716214-5 |  |
| ISC 194, 197a, 199a, 200 | *Phaetabanus fervens* * | BR/PP | 19°57’S 57°01’W | MG680252-5 | MG680212-5 |
| ISC 201, 203a | *Phaetabanus fervens* * | BR/PP | 19°57’S 57°01’W | MG680256-7 |  |
| ISC 205a | *Phaetabanus fervens* * | BR/PP | 19°57’S 57°01’W |  | MG680216 |
| ISC 206, 207a | *Phaetabanus fervens* * | BR/PP | 19°57’S 57°01’W | MG680258-9 | MG680217-8 |
| ISC 217, 218 | *Atylotus* sp. ^#^ | BR/AM | 8°56'S 62°03'W | MH716221-2 |  |
| TCC 624 | *Caiman yacare* | BR/PP | 19°57’S 57°01’W | EU596253 | EU596257 |
| TCC 625, 1100 | *Caiman yacare* | BR/PP | 19°57’S 57°01’W | KF546506 | EU596259 |
| TCC 1092 | *Caiman yacare* | BR/PP | 19°57’S 57°01’W | EU596258 | EU596254 |
| TCC 1101, 1102, 1119 | *Caiman yacare* | BR/PP | 19°57’S 57°01’W | KF546507-9 | EU596261-3 |
| TCC 1120 | *Caiman yacare* | BR/PP | 19°57’S 57°01’W | KF546510 | EU596255 |
| TCC 1829 | *Caiman crocodilus* | BR/AM | 7°27’S 64°80W | KF546511 | KF546521 |
| *T. ralphi* (TCC 1838) | *Melanosuchus niger* | BR/AM | 7°27’S 64°80W | KF546512 | KF546527 |
| TCC 1974 | *Caiman yacare* | BR/PP | 19°57’S 57°01’W | KF546513 | KF546522 |
| TCC 2218 | *Caiman crocodilus* | BR/AT | 7°32’S 49°22’W | KF546514 | KF546523 |
| **Clade Cay03** | | | | | |
| BSC 31 | *Melanosuchus niger* | BR/AM | 2°19’S 65°70’W | MG680245 |  |
| BSC 32 | *Melanosuchus niger* | BR/AM | 2°19’S 65°70’W | MG680246 | KP768298 |
| BSC 33c2 | *Melanosuchus niger* | BR/AM | 2°19’S 65°70’W |  | KP768299 |
| BSC 35c2 | *Melanosuchus niger* | BR/AM | 2°19’S 65°70’W | MG680247 | KP768300 |
| BSC 39 | *Melanosuchus niger* | BR/AM | 2°19’S 65°70’W |  | KP768301 |
| BSC 43c2*, 44 | *Melanosuchus niger* | BR/AM | 2°19’S 65°70’W | KP768279-80 | KP768302-3 |
| BSC 46 | *Melanosuchus niger* | BR/AM | 2°19’S 65°70’W |  | KP768304 |
| BSC 47 | *Melanosuchus niger* | BR/AM | 2°19’S 65°70’W | MG680248 |  |
| BSC 49c2* | *Melanosuchus niger* | BR/AM | 2°19’S 65°70’W | KP768281 |  |
| BSC 50c3*, 51c3*, 52 | *Melanosuchus niger* | BR/AM | 2°19’S 65°70’W |  | KP768305-7 |
| BSC 55 | *Melanosuchus niger* | BR/AM | 2°19’S 65°70’W | KP768282 | KP768308 |
| BSC 57c3, 58 | *Melanosuchus niger* | BR/AM | 2°19’S 65°70’W | MG680250-1 |  |
| ISC 192a, 194b, 198a, 199 | *Phaetabanus fervens* * | BR/PP | 19°57’S 57°01’W |  | MG680222-5 |
| BSC 363 | *Caiman crocodilus* | BR/AM | 8°80’S 63°95’W | KP768283 |  |
| BSC 452 | *Caiman crocodilus* | VE/OR | 6°83´N 67°69´W |  | KR107954 |
| **Clade Tab01** | | | | | |
| ISC 198b, 206a, 207b | *Phaetabanus fervens* * | BR/PP | 19°57’S 57°01’W | MG820265-7 |  |
| ISC 219 | *Glossina pallidipes* ^§^ | UG/- | 0°01'S 30°11'E | MH716223 |  |
| **Clade Grayi** | | | | | |
| BAN 1 | *Glossina palpalis* ^§^ | GA/- | - | AJ620258 | AJ620546 |
| ANR 4 | *Glossina palpalis* ^§^ | GA/- | - | AM503352 | AJ005278 |
| CroCamp 1 | *Crocodylus niloticus* | CM/- | - | FM164795 | KF546526 |
| **Clade clandestinus** | | | | | |
| *T. clandestinus* (BSC 386) | *Caiman yacare* | BR/PP | 19°57’S 57°01’W | KP768260 | KP768285 |
| BSC 29c1* | *Caiman yacare* | BR/PP | 19°57’S 57°01’W | KP768252 | KP768284 |
| BSC 388 | *Caiman crocodilus* | BR/AT | 7°66’S 49°29W | KP768262 | KP768286 |
| TSC 03 | *Haementeria* sp. ^†^ | BR/PP | 19°57’S 57°01’W |  | KP768287 |
| TSC 05 | *Haementeria* sp. ^†^ | BR/PP | 19°57’S 57°01’W | KP768263 | KP768288 |
| TSC 62 | *Haementeria* sp. ^†^ | BR/AT | 7°66’S 49°29W | KP768269 | KP768289 |
| TSC 64 | *Haementeria* sp. ^†^ | BR/PP | 19°57’S 57°01’W | KP768270 | KR107951 |

*, South American tabanid; #, African tabanid; §, Tsetse fly; †, Leech

BR, Brazil; CM, Cameroon; ET, Ethiopia; GA, The Gambia; GB, Guinea-Bissau; MZ, Mozambique; UG, Uganda; VE, Venezuela

Hidrographic basins: PP, Paraguay/Paraná; OR, Orinoco; AM, Amazonas; AT, Araguaya/Tocantins

TCC, Codes of cultures cryopreserved at the Trypanosomatid Culture Collection of University of São Paulo;

BSC, Blood Sample Collection; ISC, Insect Sample Collection; TSC, Tissue Sample Collection.

All Genbank accession numbers initiated by MG- and MH- represent sequences determined in the preset study.
